# ARTIE: An Integrated Environment for the Development of Affective Robot Tutors

**DOI:** 10.3389/fncom.2016.00077

**Published:** 2016-08-03

**Authors:** Luis-Eduardo Imbernón Cuadrado, Ángeles Manjarrés Riesco, Félix De La Paz López

**Affiliations:** ^1^SOPRA SteriaMadrid, Spain; ^2^Department of Artificial Intelligence, Universidad Nacional de Educación a DistanciaMadrid, Spain

**Keywords:** affective robot tutors, affective educational recommender systems

## Abstract

Over the last decade robotics has attracted a great deal of interest from teachers and researchers as a valuable educational tool from preschool to highschool levels. The implementation of social-support behaviors in robot tutors, in particular in the emotional dimension, can make a significant contribution to learning efficiency. With the aim of contributing to the rising field of affective robot tutors we have developed ARTIE (Affective Robot Tutor Integrated Environment). We offer an architectural pattern which integrates any given educational software for primary school children with a component whose function is to identify the emotional state of the students who are interacting with the software, and with the driver of a robot tutor which provides personalized emotional pedagogical support to the students. In order to support the development of affective robot tutors according to the proposed architecture, we also provide a methodology which incorporates a technique for eliciting pedagogical knowledge from teachers, and a generic development platform. This platform contains a component for identiying emotional states by analysing keyboard and mouse interaction data, and a generic affective pedagogical support component which specifies the affective educational interventions (including facial expressions, body language, tone of voice,…) in terms of BML (a Behavior Model Language for virtual agent specification) files which are translated into actions of a robot tutor. The platform and the methodology are both adapted to primary school students. Finally, we illustrate the use of this platform to build a prototype implementation of the architecture, in which the educational software is instantiated with Scratch and the robot tutor with NAO. We also report on a user experiment we carried out to orient the development of the platform and of the prototype. We conclude from our work that, in the case of primary school students, it is possible to identify, without using intrusive and expensive identification methods, the emotions which most affect the character of educational interventions. Our work also demonstrates the feasibility of a general-purpose architecture of decoupled components, in which a wide range of educational software and robot tutors can be integrated and then used according to different educational criteria.

## 1. Introduction

Robotics has attracted great interest among teachers and researchers as a valuable tool for developing cognitive and social skills of students, from kindergarten to middle-level education, supporting science, mathematics, technology, computing, and other subjects as well as interdisciplinary learning activities (Alimisis, 2013). In addition, it has been shown that the implementation of social support behaviors in robot tutors increases efficiency in student learning (Saerbeck et al., 2010).

The affective dimension of learning is particularly crucial in the early stages of education. Thus, in Riggs et al. (2006) the authors state that in early childhood emotional development is prior to the development of thought, and should be integrated with language and cognitive skills which develop more slowly. As set forth in Pellegrini and Blatchford (2000), in primary education social interaction among peers and with the teachers is particularly important. Taking into account the emotional dimension of learning in the design of robots, or other interfaces of educational systems for children, actively contributes to the fulfillment of learning objectives. One of the main requirements of a robot tutor must be the ability to recognize and express emotions.

In the last decade, the rise of the semantic web and online educational systems has led to standardization proposals of competency models, student profiles and learning objects among others, with the aim of representing the knowledge contained in web resources in a form which is available for applications (Gascueña et al., 2004), particularly through ontologies (Kaur and Chaudhary, 2015). The interest of affective educational systems has also motivated the formalization of models of emotions by means of ontologies and markup languages, as in the case of Bertola and Patti (2013), Grassi (2009), Hastings et al. (2012), and Hastings et al. (2011) where models are defined in terms of the ontologies HEO (Human Emotion ontology), MFO-MS (Mental Functioning ontology—Emotion ontology), or EMO (Emotion ontology).

To our knowledge, in the field of online educational systems, and specifically in affective educational recommender systems, the idiosyncrasies of primary education have not been taken into sufficient account. In particular, no references have been found to specific ontologies for modeling the emotions of primary school pupils, nor the potential of affective robot tutors been exploited by using non-intrusive low-price methods for detecting the emotional states of learners, thereby enabling them to be widely used in schools.

There are two types of methods for detecting the emotional states of students who interact with an educational software: physiological methods and behavioral methods. Resch et al. (2015), Picard et al. (2001), and Bailenson et al. (2007) report on detecting emotions based on physiological parameters measured by intrusive devices such as bluetooth bracelets or haptic devices for measuring pulses, variation of pulses, skin temperature, and others. These devices have the disadvantage that they are not cheap and that, depending on the physiological information to be read, it may be necessary to use more than one device at a time, which may cause some discomfort to the students. These factors are particularly relevant in young students.

As current robot tutors lack the data from the student's interaction with the computer which an affective educational software can access to diagnose affective states (mainly inputs from keyboard and mouse, and facial images which are easy to interpret when the student looks at the screen), they cannot exhibit such a complex social behavior as a virtual agent, unless the affective state of students is evaluated by intrusive and costly methods. However, as concluded in Saerbeck et al. (2010) the social interaction between students and robots is better than that between student and virtual agents.

Based on the above, the aim of the work reported on here is to facilitate the implementation of affective robot tutors that communicate with educational software designed for primary school children, and that take into account the idiosyncrasies of these students. Such robots will play the role of virtual advisers in an affective educational software system, that is, they will be able to identify the emotional state of students and give them consistent pedagogical support.

For this purpose, we provide an architectural pattern, a development methodology which involves the active participation of teachers, and a software development platform for the development of robot tutors which can be used with a wide range of educational software tools and commercially available robots, and under any pedagogical approach.

The rest of the paper is structured as follows. In Section 2, we present the research context and the technological choices, and we explain the user experience carried out and the methodology followed to develop an “Emotional model” component for the ARTIE platform. In Section 3 we report on the results of our research: we detail the ARTIE architecture, development methodology and platform for implementing affective robot tutors that communicate with an educational software for elementary school children; and we describe the development and evaluation of a prototype affective robot tutor, MONICA, using the educational software Scratch and the NAO robot. Finally, in Section 4 we present a discussion of our work and outline some future research lines suggested by our experience.

## 2. Materials and methods

### 2.1. Research context and technological choices

Our work can be placed in two research areas:

- The area of affective robot tutors, which have already been used in numerous studies (therapy with children with autism spectrum disorders (ASD) (Ismail et al., 2012), English classes in primary school (Keren et al., 2012), presentations of course contents in primary school (Nozawa et al., 2004), etc., and as shown in Saerbeck et al. (2010), improve the efficiency of learning, making students learning more and being more motivated.
- The area of affective recommender systems, area which, as described in Salmeron-majadas (2014), is of great importance in education, due to the strong relationship between emotions and mental processes of cognitive nature.

In this section, we present the current state in the research areas of interest for this paper, putting the work in context and justifying the technological choices taken to carry it out. Such areas are: affective robotics in education, requirements engineering for the development of affective educational software, recognition of emotional states from keyboard and mouse interactions, and markup languages for the specification of robots and virtual agents.

#### 2.1.1. Affective robotics in education

Human Robot Interaction (HRI), and, in particular, Socially Assitive Robotics (SAR) are keystones in actual robotics research. In this context, developing virtual agents as a teaching resource, given their potential to simulate real social interaction, is today an open question. However, as set forth in Saerbeck et al. (2010) the appearance of the educational agent has a significant impact on user behavior. It has been shown that having the perception of a partner to interact with can be improved by using a physical robot. Nevertheless, it should be noted that the acceptance of the robot by teachers is vital to ensuring its benefits as an education assistance tool (Fridin and Belokopytov, 2013).

Educational robots have positive influence on the learning processes, helping students to get better test scores (Catlin and Robertson, 2012) and increasing more than books or other audio-visual resources their interest (Mubin et al., 2013). Within education, robotics initially adopted the perspective of constructivism, in which students learn to solve problems by building a physical artifact. Later the notion of social constructivism proposed by Vygotsky was introduced and is now the perspective adopted by most education methodologies based on robot tutors (Mubin et al., 2013).

In primary education a large number of studies with different robots have been carried out. Two different approaches can be considered: one where the robot tutor plays the role of a teacher, teaching lessons to the student, as in Keren et al. (2012) and other where the role of the robot is to receive care, as in Tanaka and Matsuzoe (2012).

Notably, in all the studies analyzed, the presence of a robot in a classroom significantly improves the learning curve of students. However, despite the initial increase in motivation of the students due to the presence of the robots, they gradually lose interest in them (Jimenez et al., 2015). Due to these findings studies on affective education were carried out in the field of robotics.

Therefore, socially interactive robots should ideally have a number of features such as the ability to express or perceive emotions, to communicate via high-level dialogue, to learn and recognize patterns of other agents, to establish and maintain social relationships, to use natural signs, to display a distinctive personality and to learn social skills (Fong et al., 2002).

As mentioned previously, SAR have great potential for developing efficient educational tools (Keren et al., 2012). In recent years there has been an increase in the development of socially interactive robots with the ability to interpret social characteristics (Vouloutsi et al., 2014) which enable them to interact naturally with humans (Salam and Chetouani, 2015). In Fong et al. (2002), an extensive study of the state of the art on socially-interactive robots at that time is presented concluding that, to give credibility to the interaction between a robot and a person, the robot has to incorporate artificial emotions and recognize human emotions; the authors draw attention to the role of speech, facial expressions and body language as highly effective methods of communicating emotions (Breazeal and Aryananda, 2002). Robots trained to identify student emotions through facial and gesture recognition can provide effective assistance to the teacher (Veena Vijayan, 2014).

In recent times, social and assistant robots have been used in many educational projects involving preschool children, one example being the use of NAO (Softbank Robotics, 2016) in Kindergarten Assistive Robotics (KAR) (Keren et al., 2012). The use of robots in special education has also been shown to be effective, particularly in education for children with ASD (Robins et al., 2004; Ismail et al., 2012).

##### 2.1.1.1. Affective robots and recommender systems

The process of acquiring knowledge is a key component for social robots, enabling them to improve their actions in a dynamic human environment. For this reason, one of the approaches to the implementation of knowledge systems in robots is to imitate the human cognitive processes involved in the interaction with different environments (Koo et al., 2011). To operate as a genuine tutor and make appropriate educational interventions, a robot must know the learning subject, the competencies of students, and the specific circumstances which create the need for each particular learning intervention. A possible means to provide a robot with this knowledge is to establish a communication between the robot and an affective educational recommender system with which the students interact.

In the context of health care progress has been made regarding the communication between robots and recommender systems, see Hammer et al. (2015) and Tang et al. (2015). Robots connected to recommender systems have also been developed in the context of business providing other services as shown in Koo et al. (2011) and Kamei et al. (2011).

However, in the context of education to date we have not found any reference of robots which operate jointly with an educational recommender system.

There are therefore enough studies supporting the hypothesis that the use of robots, and particularly affective robots, in education can improve learning processes, as concluded in Saerbeck et al. (2010), Capponi et al. (2010), Jimenez et al. (2015), and Keren et al. (2012). The greatest benefits of using robots as an educational tool are obtained in infant and primary school children. The availability of non-intrusive and low cost methods and tools to facilitate social interaction between children and robots is crucial to the use of affective robot tutors becoming widespread in infant and primary education. Also noteworthy is the lack of research with regard to the integration of educational recommender systems and robots.

Under the above considerations, we aim to facilitate the implementation of affective robot tutors which operate jointly with an educational recommender system for elementary school children, taking into account the idiosyncrasies of these students. Such robots will play the role of virtual advisers in an affective educational recommender system, that is, they will be able to identify the emotional state of students by non-intrusive, low cost means and to provide pedagogical support accordingly. Given the importance of robot tutors as teaching tools we propose a development methodology which regards the active participation of teachers, in order to ensure consistency of pedagogical criteria and approaches.

#### 2.1.2. Requirements engineering for the development of affective educational software

With the aim of analyzing the emotional states of pupils and the pedagogical interventions of teachers, in addition to collecting both keyboard and mouse interaction data, and desktop and webcam records, other observation methods for the elicitation of knowledge during the user experience sessions were needed. These methods provide additional information for the labeling of affective states, information which must be synchronized with data logs obtained from the educational software that the students participating in the experience are using (Ocumpaugh, 2012).

##### 2.1.2.1. Observer types

Observers may be the students themselves, who can list the emotions first hand experienced by self-reports (as in the case of SAM), outside observers belonging to the educational environment (teachers or students) or external observers who do not belong to that environment (Porayska-Pomsta et al., 2013). In our case, since teachers were busy involved in the educational task, external observers were placed in strategic locations in the classroom so that they did not interfere with the experiment.

##### 2.1.2.2. Methods of recognition of student affective responses

The purpose of these methods is to identify the emotional states which are associated with different learning circumstances. Annotations of these emotional states are performed during the observation sessions. The recording of these emotion states can be performed in real time or retrospectively. External annotators can observe how students perform tasks in real time and describe their emotional states (Ocumpaugh and College, 2014). For external annotators there is also the possibility of making observations retrospectively, by viewing videos and making annotations of the affective states some time later (Ocumpaugh and College, 2014).

In our experiment we made general recordings of the sessions for further analysis. However, we also found it of interest to make annotations in real time during the sessions, since in video recordings some aspects of the recorded scenes are lost, and some nuances of the emotional atmosphere can only be perceived or sensed face to face. For this reason we have followed the BROMP method (Baker-Rodrigo Ocumpaugh Monitoring Protocol) described in Porayska-Pomsta et al. (2013). This method was designed to study the behaviors and emotions in computer-based learning environments. A particularly relevant study for this project is Ocumpaugh and College (2014) where the use of a tool called HART (Human Affect Recording tool), an Android application designed for making annotations of affective student responses following the BROMP method, is discussed.

##### 2.1.2.3. Methods for identifying affective pedagogical interventions

The objective of these methods is to determine the appropriate affective pedagogical responses according to the emotional state of the students and the learning circumstances. A study of particular relevance to this project is (Blazar, 2015), the purpose of which was to design a new method for evaluating teachers. Two evaluation methods were implemented: CLASS (Classroom Assessment Scoring System) and MQI (Mathematical Quality of Instruction). To apply these methods, video recordings of the classes were conducted and subsequently analyzed. This has been the procedure followed in our research.

In this context, mention may also be made of the TORMES methodology. This methodology is based on the ISO standard 9241-210 and aims to involve educators in the process of designing educationally oriented recommendations (Santos and Boticario, 2011) (Manjarrés-Riesco et al., 2013).

#### 2.1.3. Recognition of emotional states from keyboard and mouse interactions

Affective measures can be grouped into three different areas: physiological, behavioral and psychological (Salmeron-majadas, 2014). The conventional methods of emotion identification (facial expression analysis, analysis of voice intonation, etc.) require intrusive, expensive tools, unpractical in real settings (Khan et al., 2013). Since the purpose of our work is precisely to facilitate the development of realistic non-intrusive and low cost educational systems which can be used in different educational contexts, we base the identification of emotions on keyboard and mouse user-interactions. This type of information input is the least intrusive and requires no special devices (Rajput and Vijayavargiya, 2015).

Emotion recognition based on log processing requires analysis of typing dynamics and mouse interactions, and the subsequent application of data mining methods.

##### 2.1.3.1. Analysing keyboard interactions

The analysis of keyboard interactions is aimed at identifying student emotional states paying attention not so much to what you type as to how you type (Khan et al., 2013). A multitude of keyboard interaction parameters have been studied in the literature in order to identify emotional states, such as in Rajput and Vijayavargiya (2015), Khanna and Sasikumar (2010), and Khan et al. (2013). In addition, different approaches to collect these parameters, as well as the student emotional states, have been described, such as in Rajput and Vijayavargiya (2015) and Khan et al. (2013).

The typing parameters considered in our research are: the total number of times the return and the delete key are pressed, key latency (interval between releasing of a key and pressing the next key) and key-press time (time during which a key is held down).

##### 2.1.3.2. Analysing mouse interactions

The analysis of mouse interactions aims to identify the emotional states of the student on the basis of his or her handling of the mouse. (Khanna and Sasikumar, 2010) shows that the combined analysis of keyboard and mouse interactions significantly improves the results. Numerous mouse parameters are analyzed in literature, such as in Zimmermann et al. (2003) and Salmeron-majadas (2014).

The mouse parameters considered in our research are: number of mouse clicks per minute, average distance between mouse clicks on the screen (from being pressed until released), total distance covered by mouse movements in pixels, number and length of pauses in mouse movement, number of abrupt movements (more than 5 changes of direction in 2 s), and maximum, minimum and average mouse speed.

##### 2.1.3.3. Emotional states

Between two and twenty “basic emotions” such as happiness, fear, love, surprise, sadness, etc. have been considered in the different approaches to emotion research (Khan et al., 2013). Complex emotions can be defined as a combination of the most basic emotions.

Student emotions or, strictly speaking, student cognitive-affective states such as trust, sadness, nervousness, happiness, indecision….and their relationship with keyboard and mouse interactions have been studied in Rajput and Vijayavargiya (2015), Khanna and Sasikumar (2010), and Bradley and Lang (1994).

After conducting the user experience we report later, and having interviewed primary school teachers who perform activities with computers, we conclude that, in the case of students in this educational phase, there are, as a first approximation, three states having significant repercussions on the nature of the appropriate pedagogical intervention. These states do not correspond to specific emotions but rather to categories of cognitive-affective states:

- Concentrating: When the student is engaged in learning activities and has an adequate interaction with the educational program. This state involves emotions of awe, curiosity, confidence, enthusiasm, interest, intrigue…
- Distracted: When the student is distracted performing tasks different from those assigned (using other programs) or his or her interaction with the educational program is irregular. This state involves emotions of boredom, apathy, indifference, laziness,…
- Inactive: The student has doubts, he is talking with peers and no interaction or minimal interaction with keyboard and mouse is registered. This state involves emotions of boredom, confusion, discouragement, anger, frustration, helplessness, irritability, pessimism…

##### 2.1.3.4. Keyboard and mouse interaction data mining for identifying emotional states

Approaches using both predictive methods (classification, regression, categorization, …) as well as descriptive methods (clusterization, correlation, rule association, …) have been proposed in the literature for the automatic identification of emotional states based on keyboard and mouse interaction data. Thus, in Salmeron-majadas (2014) C4.5, Naive Bayes, Bagging, Random Forest, and AdaBoost algorithms have been used, while in Rajput and Vijayavargiya (2015) discriminant analysis methods, Bayesian analysis, k-neighbors, artificial neural networks and decision trees are the chosen methods.

In Cocea and Weibelzahl (2009) the k-neighbor method provided very good results. Decision trees (C4.5) have also been shown to be successful in Salmeron-majadas (2014). Likewise, artificial neural networks and Nave Bayes have been implemented in Salmeron-majadas (2014) (Rajput and Vijayavargiya, 2015) showing their effectiveness.

Despite the many studies on this subject, we still found it of interest to examine the particular case of primary school pupils, students who interact in a particular way with computers (they make more sudden and random movements, get easily discouraged…). In the research work reported on here we have experimented with the aforementioned data mining methods and, additionally, with the Support Vector Machines method, a classification method appropriate in the case of high dimensionality which has never before been applied in this field.

#### 2.1.4. Markup languages for the specification of robots and virtual agents

Markup languages are used, among other things, to defining the structure of documents with the aim of exchanging information between different systems without specifying how this information should be treated. The languages of interest in the present technological study are those suitable for specifying either robots or virtual avatars, including the representation of affective states, such as the XML (eXtensible Markup Language) based languages RoboML (Shelton, 2012; Choi et al., 2014), AIML (Gocłowska et al., 2007; Liu and Dong, 2015), VHML (Beard et al., 2001; Prendinger et al., 2011), MPML (Tsutsui et al., 2000), EmotionML (Meftah, 2013), FML (Functional Markup Language), and BML (Behavior Markup Language) (Heylen et al., 2008).

FML and BML are complementary markup languages designed to represent an agent strategy and behavior. FML allows describing what the agent has to perform, including gestures and oral expressions, without specifying how (Ribeiro et al., 2013). Reciprocally, BML is used to specifying how an agent has to perform the actions previously described in FML (Kopp et al., 2006).

FML and BML are both independent from the virtual physical platform (robot or virtual avatar), as presented in Paiva et al. (2012). The results presented in Ribeiro et al. (2013), regarding an empathetic robot tutor which plays a collaborative game with students, are of special relevance to our research. The same applies to the research work described on (Lohse and Welbergen, 2012), where behaviors are transferred between robots (including NAO) and virtual agents.

In this article we propose the implementation of the BML markup language to specify and exchange information about the behavior of the robot (movements, gestures and dialogues, specifying tone, volume of voice…), with special emphasis in the expression of emotions.

### 2.2. User experience with scratch

The purposes of the user experience related below were:

- Collecting data to assist the development of a component of the ARTIE platform for identifying student emotional states through the use of data mining techniques. This component may be used with a wide range of educational software tools such as Scratch.
- Elicitating pedagogical expert knowledge from primary school teachers for the design of a general framework, another component of the ARTIE platform, where adequate affective pedagogical interventions in the presence of different learning scenarios are specified.
- Orienting the definition of the ARTIE development methodology.
- Defining the specific requirements for the development of a prototype of MONICA, an affective robot tutor based on NAO for tutoring primary school students practicing with Scratch.

Scratch is a programming language primarily target to children which aims to make them better understand the programming concepts by allowing to create interactive histories, animations, games, and even music and art compositions, through palette blocks organized into sections (Valle and Salgado, 2013). Scratch has been integrated in academic curricula in many countries, such as Mexico (Valle and Salgado, 2013), United Kingdom (Wilson and Moffat, 2010), Turkey (Kalelioglu and Gülbahar, 2014), or Colombia (López García, 2012).

As stated in previous sections, we wanted to base the recognition of cognitive-affective states on the analysis of keyboard and mouse interactions, a non-intrusive and low-cost identification method that, in the case we are addressing, brings quite good results. Given that our work is oriented to primary school students and that we found no reference to databases of keyboard and mouse interaction data tagged with cognitive and affective state labels for such students, we decided to compile this data by recording learning sessions with Scratch during a primary school class.

Recording sessions were held at Los Peñascales' school of Las Rozas (Madrid). The experiment involved two groups of 10 students each, aged between 10 and 11 years (5th course of primary school). Each group participated in two recording sessions of 45 min per session. During the recording sessions a fixed camera was placed in a classroom's corner, so that the whole scene was recorded and every teacher's pedagogical intervention was captured. In order to collect all data from keyboard and mouse interactions of every computer used by the students, a specific software was required. The following requirements were considered for the development of this software:

- Keyboard and mouse interactions must be registered in a standard format (we chose a CSV format).
- With respect to recording user interaction data, interactions with Scratch must be distinguishable from interactions with any other software (navigators, games, etc).
- In order to analyse and label the content of the user-interaction data logs it must be correlated with the cognitive-affective states. To help identifying these states webcam videos and screen capture videos showing the students actions at all times were also recorded.

The software was designed to be independent from the Scratch educational tool in order to be suitable for a wide range of educational software tools which operate via keyboard and mouse interactions. The implementation language was Java, so that it could be executed in any operating system, and a user-friendly interface was designed taking into account the characteristics of primary school students. This interface enables the students to identify themselves and to initiate the recording process pressing a start button, thus begining the generation of the CSV file where logs and videos (webcam and screen capture videos) are registered. The recording can be stopped by pressing the stop button.

In order to register the pedagogical interventions associated to the various learning circumstances, three external observers were taking notes by using a questionary designed following the BROMPT methodology.

**Figure 1:**
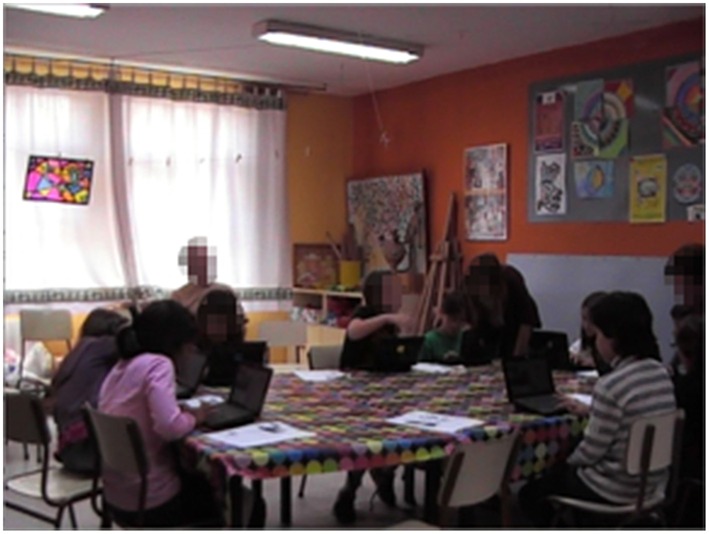
**Scratch user experience frame**. Reproduced with permission from the adults and the parents of the children.

This application has enabled us to collect data in four recording sessions. On Figure 1 a frame from a video recorded in a session is shown.

### 2.3. Development of the emotional model component

#### 2.3.1. Description of the sample

We have worked with a sample of the keyboard and mouse interaction data from 20 students between 10 and 11 years old, in 4 sessions of 45 min each. The size of the log file of each student varies depending on the actual duration of the session and the number of interactions realized during the sessio. In the Table 1 the sample data used is shown.

**Table 1:** **Sample data by concentration states**.

**Affective-cognitive states**	**Number of instances**
Concentrating	670
Distracted	600
TOTAL	1359

#### 2.3.2. Attribute selection

The attribute selection has four main objectives. First, it allows to reduce the data size. Secondly, it can improve the quality of the model, making it to be focused in the relevant characteristics. Thirdly, having less attributes, the model reading ca be performed by decision trees, lineal regression models, etc. Finally attribute selection also facilitates the understanding of the data visually (Hernández Orallo et al., 2004). In our case, the most relevant objective is to improve the quality of the model by eliminating the less relevant characteristics.

The Table 2 represents the attributes that are used as input for the selection and evaluation methods.

**Table 2:** **Attributes of the input for the selection and evaluation methods**.

**ID**	**Attributes**	**Description**
1	BackspaceKeys	Number of times the backspace key is pressed
2	DeleteKeys	Number of times the delete key is pressed
3	LatencyKeys	Key latency
4	PresstimeKeys	Key-press time
5	ClicksMouse	Number of mouse clicks
6	MeanDistanceClicksMouse	Average distance between mouse clicks in pixels
7	PauseMovementMouse	Number of pauses in mouse movement
8	PauseMovementSizeMouse	Length of pauses in mouse movement
9	AbruptMovementMouse	Number of abrupt movements
10	MaxSpeedMouse	Maximum mouse speed
11	MinSpeedMouse	Minimum mouse speed
12	MeanSpeedMouse	Mean mouse speed
13	CLASS	Sample classification

There are two different methods for attribute selection: The attribute selection methods, that provide a core set of significant attributes, and the evaluation methods that provide an attribute ranking from the most to the less significant ones. Both of them are complementary.

In Table 3 there are the results of the different attribute selection and evaluation methods analyzed in this study.

**Table 3:** **Results table of the attribute selection and evaluation methods**.

**Selection methods**	**Filter/Model**	**Options**	**Strategy**	**Selected attributes**
CfsSubsetEval	Filter	Default values	BestFirst	1,2,4,5,6,7,8,9
		J48	BestFirst	2,3,4,5,6
WrapperSubsetEval	Model	BayesNet	BestFirst	3,4,5,6,9,12
		SMO	BestFirst	1,4,5,6,7,9,10,11
**Evaluation method**	**Filter/Model**		**Strategy**	**Attribute order**
InfoGainAttributeEval	Filter		Ranker	7,5,8,10,12,4,3,9,6,1,11,2
ReliefFAttributeEval	Filter		Ranker	8,7,3,4,6,12,10,1,5,9,2,11

Because the attributes handled are continuous, we selected the methods that have a good behavior with this kind of data. In Table 4, the resulting table of the different attribute selection and evaluation methods is shown.

**Table 4:** **Attribute selection of the sample data**.

**ID**	**Attributes**	**Description**
3	LatencyKeys	Key latency
4	PresstimeKeys	Key-press time
5	ClicksMouse	Number of mouse clicks
6	MeanDistanceClicksMouse	Average distance between mouse clicks
7	PauseMovementMouse	Number of pauses in mouse movement
8	PauseMovementSizeMouse	Length of pauses in mouse movement
9	AbruptMovementMouse	Number of abrupt movements
10	MaxSpeedMouse	Maximum mouse speed
12	MeanSpeedMouse	Mean mouse speed
13	CLASS	Sample classification

Although it appears in the SMO selection method, the *MinSpeedMouse* attribute is not a relevant attribute, because in any of the affective-cognitive states the minimum speed of the mouse can be zero (for example, if the student is writing and the mouse is not in movement). Nevertheless in the evaluation methods this parameter (11) appears in the last or penultimate relevant position.

Two attributes that are not relevant in this study are those that indicate the pressing of specific keys [*BackSpaceKeys* (1), *DeleteKeys* (2)], because the pressing of these keys does not indicate anything about the concentration states, and the key-press time can be read with the attributes *LatencyKeys* (3) and *PressTimeKeys* (4).

In the evaluation method InfoGainAttributeEval it can be seen that these attributes (1 and 2) are in the last position of the ranking.

The Table 4 shows the attributes that were finally used for the clustering and classification methods.

#### 2.3.3. Classification model

In this section we evaluate different classification models.

As we have seen in Section 2.1.3, for the automatic identification of emotional states based on keyboard and mouse interaction data, there are two different data mining methods used in the literature: predictive methods (classification, regression, categorization, …) and descriptive methods (clusterization, correlation, rule association,…).

In one hand, within the predictive methods, in the literature authors make use of: C4.5, Naïve Bayes, Bagging, Random Forest, AdaBoost, artificial neural networks and Bayesian analysis.

As we have seen in Section 2.1.3, because of the good results provided in different studies, the C4.5, Naïve Bayes and artificial neural networks are chosen to be analyzed. Moreover, these methods have the peculiarity that they have a good behavior with continuous attributes, as is our case.

In the other hand, within the descriptive methods, in the literature, authors make use of: discriminant analysis methods and k-neighbors.

Because of the good results provided in different studies, the k-neighbors is chosen to be analyzed.

In addition to these methods, the SVM method will be analyzed because it exhibits a good behavior with continuous attributes, and it works fine with high dimensionality that separates with hyperplanes.

#### 2.3.4. Naïve bayes

Because of the good results of Naïve Bayes, explained in Section 3, we have implemented this method to generate the classification model.

The main basis of Naïve Bayes (or NB) is the assumption that all the attributes are independent when the class value is known. This hypothesis of independence assumed by the NB classifier results in a probabilistic graphical model in which there is only one root node (the class), and in which all attributes are leaf nodes whose sole parent node is the class (Hernández Orallo et al., 2004).

**Figure 2:**
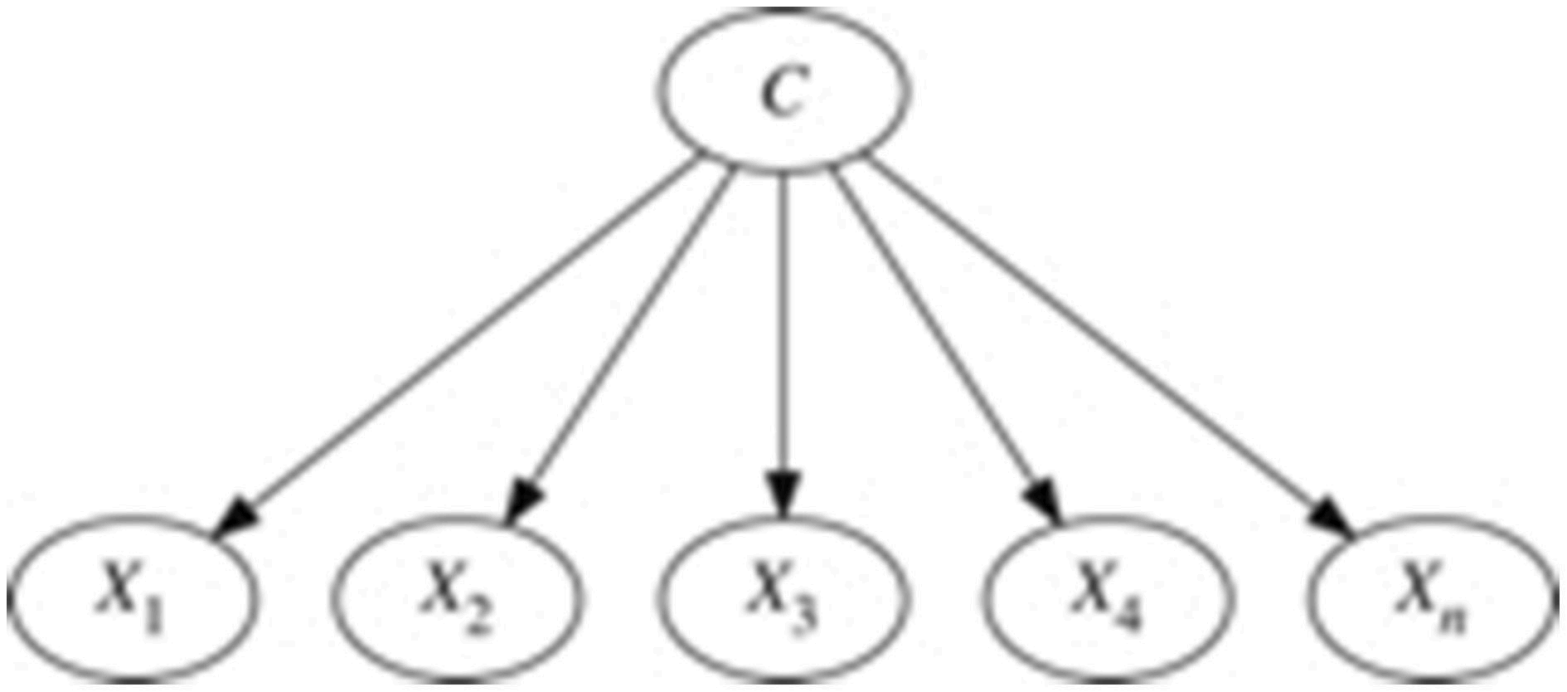
**Topology of a Naïve Bayes classifier**.

In Figure 2 the topology of a Naïve Bayes classifier is shown.

Because of the assumption of independence used in Naïve Bayes, the expression to obtain the MAP hypothesis is as follows:

(1) $$\begin{array}{r} c_{MAP}=\arg\underset{c\in \Omega_{c}}{\max}p\left(c\right)\underset{i=1}{\prod }p\left(A_{i}|c\right) \end{array}$$

Therefore, the parameters to be estimated are *P*(*A*_*i*_|*c*) for each attribute and the a priori probability *P*(*c*) class variable.

Depending on whether the *A*_*i*_ attribute is discrete or continuous, the estimate is made differently. In our case, the attributes are all continuous, so the classifier NB assumes that the attributes follow a normal distribution, and the only thing that should be calculated is the mean μ and standard deviation σ conditional on each value of the variable class.

(2) $$\begin{array}{r} P\left(A_{i}|c\right)\propto N\left(\mu ,\sigma \right)=\frac{1}{\sqrt{2\pi .\sigma }}exp\left(-\frac{{\left(X-\mu \right)}^{2}}{2\sigma^{2}}\right) \end{array}$$

Being *c* the class value and *n*_*c*_ the number of instances for this class in the dataset, the calculation of the mean μ for the attribute *A* is as follows:

(3) $$\begin{array}{r} \mu =\frac{\overset{n_{c}}{\underset{i=1}{\sum }}A_{i}}{n_{c}} \end{array}$$

And finally, based in the previous variables, the calculation of the standard deviation σ for the attribute *A* is as follows:

(4) $$\begin{array}{r} \sigma =\sqrt{\frac{\overset{n_{c}}{\underset{i=1}{\sum }}{\left(A_{i}-\mu \right)}^{2}}{n_{c}-1}} \end{array}$$

## 3. Results

### 3.1. ARTIE architecture, development methodology, and platform

In this section we describe the different components of the ARTIE architecture, as well as the ARTIE development methodology and platform for the development of affective robot tutors that communicate with an educational software tool for primary school children.

#### 3.1.1. ARTIE architecture

The ARTIE architecture is a reactive-deliberative robot architecture involving both the situated, the connectionist and the symbolic artificial intelligence paradigms. The robot interacts with its environment in real time, identifying the students by sensors, and communicating with them through effectors (via spoken language and body gestures, with emphasis on expressing the affective dimension), depending on its changing perceptions (with emphasis on perceiving the student emotional states), either reactively or deliberatively.

**Figure 3:**
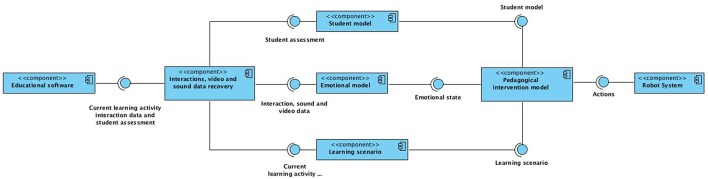
**ARTIE architectural pattern**.

On Figure 3 the ARTIE architectural pattern is shown.

The components of this architecture are:

- **Educational Software:** This component corresponds to the software with which the student interacts.
- **Interactions, video and sound data recovery component:** This component receives from the educational software all data about students and learning activities, and gathers all the student-computer interaction data, as well as the video and/or sound data being recorded. Within the framework of a reactive-deliberative paradigm this component is part of the perception system. This component also contains a part of the robot short-term memory, where the current learning activities, interactions, video and/or sound changing data are registered.
- **Student model:** This component represents the student, initially on the basis of his or her academic background and personal characteristics (learning style, skills, etc). It receives from the educational software the *assessment of the student learning processes*, and returns *updated student models*. In the frame of a reactive-deliberative paradigm, this component is part of the reasoner, since the assessment data are dynamically updated in order to establish an action plan (a tutoring strategy). This component also contains a part of the long-term memory of the robot where the more permanent characteristics of the students are stored.
- **Emotional model:** This component receives the *interaction*, *video*, *and*/*or sound data* and returns an *emotional state identified* on the basis of the keyboard and mouse interaction data, and/or from of a set of features extracted from the videos (facial and gestural analysis parameters, etc) and from the sound records (speech analysis parameters, ambient noise analysis parameters, etc). In the frame of a reactive-deliberative paradigm, this component is part of the perception system and part of the reasoner system since it is intended to identify, based on perceptions, the emotional states that will determine the action plan, that is to say, the strategy for pedagogical interventions (either in a reactive or deliberative mode, as appropriate). This component contains also a part of the short-term memory where the interactions, video and sound data are stored.
- **Learning scenario:** This component receives the *data related to the specific learning activity* being currentely performed by the student and returns a *specification of the learning scenario* involved.
- **Pedagogical intervention model:** This component receives the *dynamic student model*, the *current emotional state*, and the *current learning scenario*, and returns the *actions* that the robot must perform.

The component includes a set of rules associating affective educational interventions to specific learning scenarios, and student profiles and emotional states. The interventions (including facial expressions, body language, tone of voice,…) are specified in terms of BML files which are translated into the actions of the robot tutor.

- **Robot system:** In the frame of the reactive-deliberative paradigm, this component corresponds to the execution system that is responsible for ordering the movements in the effectors. This component contains a part of the short-term memory, where the student identifiers and the data obtained by the robot sensors are stored.

Part of the identified components are the usual ones of an affective educational recommender system, as proposed in Santos et al. (2014).

The functionality of these components will be enhanced in future versions of the ARTIE environment.

#### 3.1.2. ARTIE development methodology

Hereafter we briefly present a set of guidelines and applicable phases for the development of affective robot tutors following the ARTIE architectural pattern:

- First phase: User experience. The elicitation of knowledge about the pedagogical interventions is a basic process for designing the affective tutoring strategy that the robot will follow when interacting with students. For this elicitation, one or more user experiences are made using the educational software in question, in order to obtain information about how and when the teacher acts, and under what circumstances, ie, what learning scenarios will require with most probability the pedagogical intervention.
  − To begin with, it is necessary to design a series of activities to be carried out with the educational software such as to give rise in students the greatest possible number of emotional states to be analyzed.
  − Regarding the recording of experiences, the sessions must be recorded in a format that facilitates the subsequent analysis of the different situations that students experience, and the actions of the teacher (usually in audio and video format). In addition to these recordings, during the sessions observers complete forms based on the BROMPT methodology, recording data on the educational interventions as well as on the attitudes and emotional responses of the students.
  − Finally, for generating a specific classification model of the emotional states, it is mandatory to develop tools for gathering data that will be analyzed to generate the model.
- Second phase (optional): Generation of the emotional state classification model through either computational learning methods or data mining methods (recall that the ARTIE development platform provides a component, the Emotional model, usable with different educational software tools).
- Third phase: Instantiation of the pedagogical intervention model. Once the recordings and annotations of the user experiences have been analyzed, the task of specifying the pedagogical interventions is facilitated (as explained in the next chapter) since the user can now perform it by parameterizing the framework provided in the development platform.
- Fourth phase: Development of the Learning Scenario component, the component for the identification of the learning scenarios. This component is specific to each educational software tool so the platform can not even offer a framework to support its development.
- Fifth phase (optional): Development of a translator from pedagogical interventions to robot actions. This module is also very specific to the robot used. Currently the ARTIE platform provides a translator for the NAO robot.

#### 3.1.3. ARTIE development platform

One of our main objectives has been to provide a generic platform for the development of affective robot tutors according to the proposed architecture. Currently the ARTIE platform provides:

- An Emotional model, usable with different educational software tools. This model receives as input parameters the keyboard and mouse interactions (as explained in Section 2.3, the input parameters can be shown in Table 4), and returns as output the cognitive-affective state of the student (concentrating, distracted or inactive).
- A generic model of pedagogical intervention (see the next section).
- A translator from pedagogical interventions to robot actions for the NAO robot.This translator receives as input parameter the specification of a pedagogical intervention in BML format and translates this specification to robot actions for the NAO robot.

In the following section we explain the generic pedagogical intervention module developed.

**A Framework for the Pedagogical Intervention Model Component**

The ARTIE architecture comprises a generic model of pedagogical intervention, responsible for generating the behavior of the robot depending on the student model and his or her emotional status.

The implementation of this model is dependent on the learning domain and on the educational software tool, so for each application that implements the ARTIE architecture, the model of the pedagogical intervention may be different.

So far we have generated a basic pedagogical intervention model containing particular rules for tutoring students learning with Scratch educational software tool, and which we intend to deepen in the future. This module has been developed based on a series of guidelines found in Hernando (2015) that depend on the motivation and competence of students. These guidelines are:

- Higher competence and Higher competence: **Tutor of inspiration**
  − Seeks to raise the interest and motivation for the task.
  − Focuses on more examples and case studies to represent the content in daily life or in other projects and areas of the school.
  − Emphasizes rewards in short periods of time.
  − Asks about the lack of connection with the content, and suggests the exploration of the possible solutions.
  − Uses puzzles and games related with the contents.
- Higher competence and Higher motivation: **Coach tutor**
  − Allows to freely experiment and make mistakes.
  − Presents content or more difficult problems that encourage new challenges.
  − Maintains the interest and wonders about linking to the content and the most satisfying parts or the favorite parts.
  − Encourages to be mentors to other peers.
  − Encourages reflective moments about the nature of the content and its usefulness.
- Lower competence and Lower motivation: **Directive tutor**
  − Concrete and simple goals for short periods of response are marked.
  − Marks concrete and simple goals for short periods of response.
  − Holds highly targeted sequences with constant rewards.
  − Maintains contact and close monitoring.
  − Seeks concrete examples of the use of the content in everyday life.
  − Proposes to repeat the same exercises on more than one occasion.
  − Aims to establish relations with strategies in other areas where there is better competition and greater motivation.
- Lower competence and Higher motivation: **Guide tutor**
  − Displays the future when the learning goals have been achieved.
  − Encourages to work in a faster way, focused on increasing the difficulty of the task.
  − Offers more tasks and activities to repeat them and make their own.
  − Stresses cognitive strategies to solve a problems.
  − Strengths motivation with goals and rewards to medium and long term.

To develop a model of specific educational intervention, ARTIE platform comprises a configurable tool to define and generate the rules of pedagogical affective interventions. An example of such rules is as follows:

**Algorithm 1:** Example of intervention rules

**if** *competent* = *TRUE AND motivated* = *TRUE* **then**
| Intervention1
end
**if** *competent* = *TRUE AND motivated* = *FALSE* **then**
| Intervention2
end

Where:

**Algorithm 2:** Example of intervention definitions

*Intervention*1: it seeks to raise interest and motivation for the task
{
*Dialog* = (here a suggestion of dialogue that must be adapted to each educational software and learning specific scenario is provided, for example: ”I've seen what you're doing. Great! Keep it up and you will become as wise as Splinter!”).
*VoiceTone* = (here a suggestion of appropriate tones is provided, such as 'cheerful tone, proud tone, etc”).
*Gestures* = (here a suggestion of appropriate gestures is provided, for example: ”lift arm”).
}
*Intervention*2: It focuses on more examples and case studies
{
*Dialog* = (here a suggestion of dialogue that must be adapted to each specific case is provided, for example: ”I'll give you an example …”)
*VoiceTone* = (here a suggestion of appropriate tones is provided, such as ”friendly tone, suggestive tone, etc”)
*Gestures* =((here a suggestion of appropriate gestures is provided, for example, ”put his hand to his head”)
}

Thus, the recordings of the experiences would serve primarily to identify the more common scenarios that require an affective pedagogical intervention for a particular educational software tool. To implement the corresponding model of pedagogical intervention, the user of the platform would simply associate each scenario with a set of rules (one for each pair of values competence—motivation) and edit the rules to suit each case. The rules are translated automatically to specifications of an affective avatar and then to robot actions without the implementer having to know the corresponding specification languages.

### 3.2. MONICA: a NAO based affective robot for tutoring with scratch

Along this section we will describe the development carried out using the ARTIE environment with SCRATCH as educational software and with NAO as robot tutor; as well as a preliminary evaluation of the developed prototype.

#### 3.2.1. Implementation with scratch and NAO

**Figure 4:**
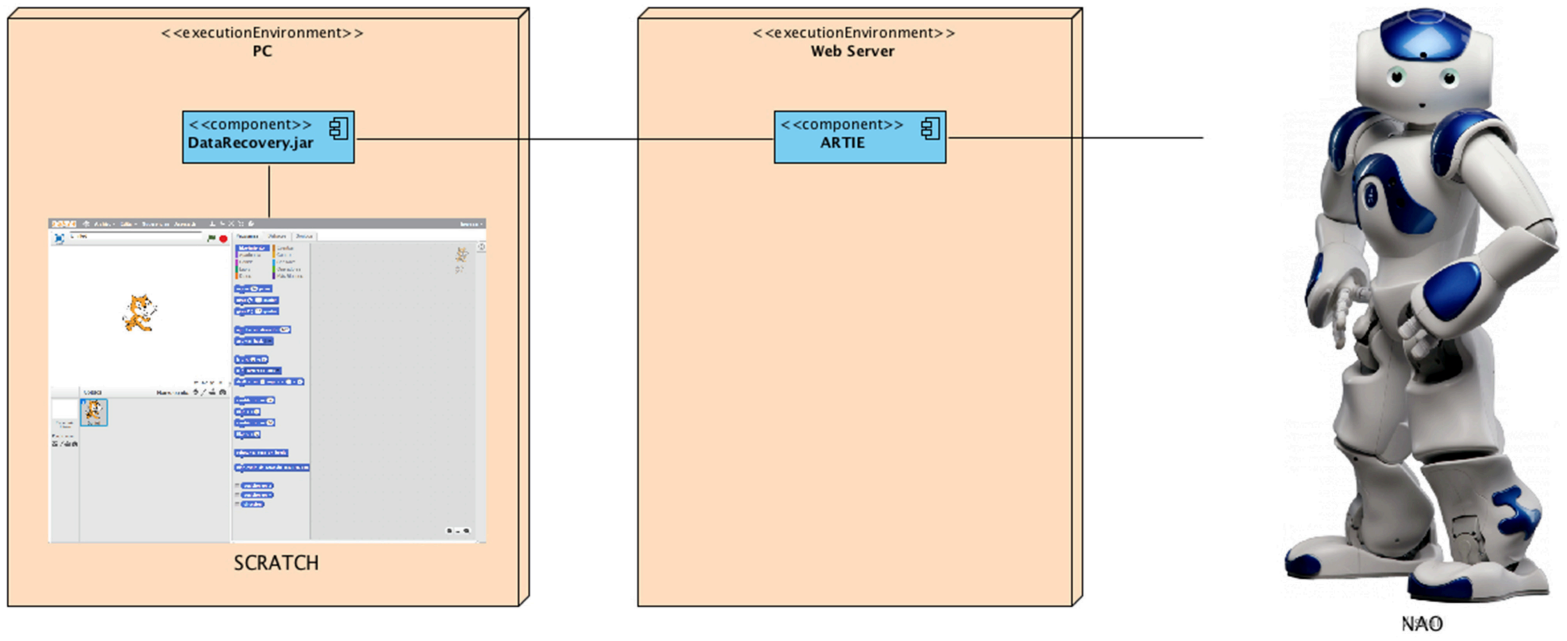
**ARTIE architecture deployment with Scratch and NAO**.

On Figure 4 the ARTIE architecture deployment with Scratch and NAO is illustrated.

To implement the ARTIE architecture in a particular physical robot the Markup Translator module, a web service responsible for translating the BML specifications into specific robot actions, should be adapted.

NAO includes an API that provides a multilingual programming environment supporting Python, C++ and Java among others. Given that the ARTIE architecture was developed in Java, we chose this language for robot function calls. Several classes were implemented in order to translate BML specifications into NAO actions.

The possible actions of NAO include motor movements (of the arms, hands, head, etc), changes in led colors (in head, ears and eyes), winks (eyes leds turning on and off), as well as TTS (Text To Speech) and STT (Speech To Text) translation among others.

A core aspect of our work, in which we will focus our future research, is the construction of the dialogues with students.

To generate a fluent dialogue and to be able to help students, when the robot detects a student's response (through STT) the Pedagogical intervention model is consulted via the Markup translator module. Additionally, this module activates in NAO the gaze toward the student, so that he or her knows at every moment if the robot is talking to him or hers.

#### 3.2.2. MONICA evaluation

The ARTIE architecture described in Section 3.1.1 has been evaluated in an experience with two primary school students. To carry out this experience, we first implemented the architecture as described in Section 3.2.1. Then, we designed some exercises for practicing with Scratch and we chose two primary students with different profiles (an 8 year old girl with high motivation and high competence; and an 11 year old boy with low motivation and high competence) and with an elementary knowledge of Scratch. The experience was recorded by a fixed camera behind the students at such a distance that we could understand the dialogue between the student and NAO.

The children performed the exercises separately, and NAO reacted appropriately to the different cognitive-affective states and the different student models. Thus, the student with high motivation and high competence was encouraged to carry on with the tasks when she got distracted for a moment. With regard to the student with low motivation and high competence, while he was performing tasks different from those assigned by the teacher the robot intervened trying to motivate his interest for the assigned tasks and to guide his practice.

**Figure 5:**
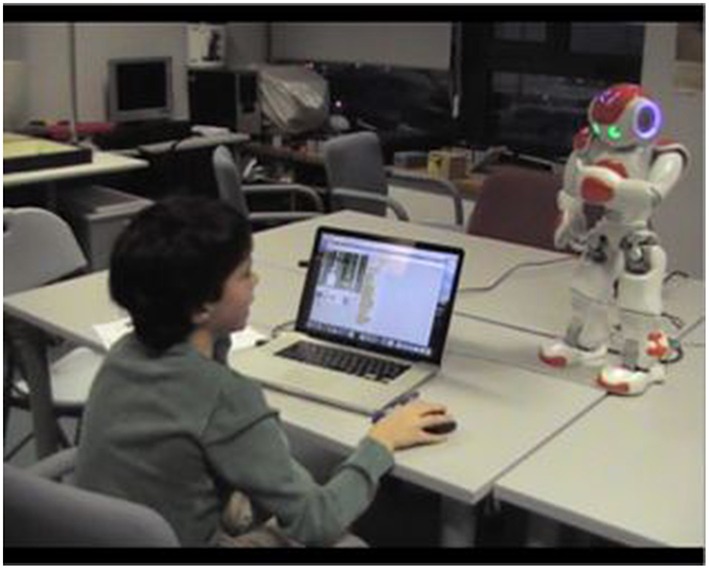
**Frame of the tutoring experience of the student with low motivation and high competence**. Reproduced with permission from the parents of the children.

**Figure 6:**
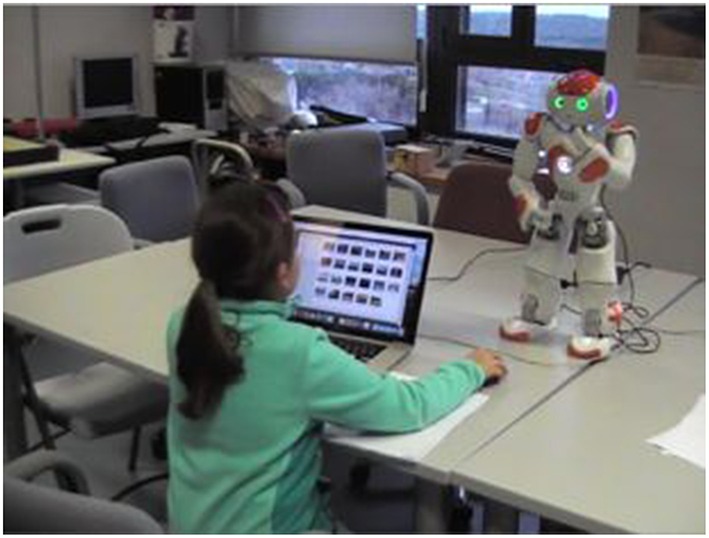
**Frame of the tutoring experience of the student with high motivation and high competence**. Reproduced with permission from the parents of the children.

The videos recorded during the experience were archived for later analysis. In Figures 5, 6 the tutoring experience is illustrated with two frames of these videos.

The participant students evaluated the experience responding to a survey designed based on Chuttur (2009), Liu et al. (2010) Pardo et al. (2009), and Selim (2003). The survey is divided into five sections whose questions have an associated Likert scale of 5 degrees being (1) “strongly disagree” and (5) “strongly agree.” Three open questions were also included at the end of the survey.

Below we show the survey answers for each student, being “Student 1” the student with high motivation and high competence, and “Student 2” the student with low motivation and high competence.

##### 3.2.2.1. Section 1: Choice of the medium/technical aspects

Assessing the choice of a robot (and of MONICA, in particular) for affective tutoring of primary school children who practice with Scratch educational software; see Table 5.

**Table 5:** **Students answers to section 1 of the survey**.

	**Student 1**	**Student 2**
I like to have a robot guide my learning	5	3
I prefer to have a robot tutor guide my learning rather than a virtual agent	3	5
I prefer a robot tutor to a human teacher	1	2
I prefer to have a robot tutor rather than to work alone	4	5
I like the MONICA robot	5	4

##### 3.2.2.2. Section 2: Robot performance

Assessing how the robot operates in practice; see Table 6.

**Table 6:** **Students answers to section 2 of the survey**.

	**Student 1**	**Student 2**
MONICA identified the situations that arose during me session with Scratch	1	1
I understood her messages well	1	2
The messages were appropriate to the circumstances	1	2
She understood my answers	1	2

##### 3.2.2.3. Section 3: Perception of usefulness

Assessing the effect that the use of the robot tutor has in learning; see Table 7.

**Table 7:** **Students answers to section 3 of the survey**.

	**Student 1**	**Student 2**
MONICA helped me with the problems I had	1	2
She has helped me to learn how to use Scratch	1	1
I have learned more than with a teacher	3	1
I feel more relaxed working than when a teacher supervises me	5	5
I learned more than I do when I work alone	3	3
I was more motivated than when I work alone	1	2
I had fun talking to her	3	3

##### 3.2.2.4. Section 4: Perception of ease of use

Assessing the usability of the educational tool; see Table 8.

**Table 8:** **Students answers to section 4 of the survey**.

	**Student 1**	**Student 2**
I find it easy to communicate with MONICA	1	2

##### 3.2.2.5. Section 5: Intention of use

Assessing the final degree of acceptance of the educational tool; see Table 9.

**Table 9:** **Students answers to section 5 of the survey**.

	**Student 1**	**Student 2**
I would like to participate in another experience of this kind	5	4
I would like to have robot tutors at school in class	5	5
I have told my classmates about the experience	1	1
I have told my teacher about the experience	1	1

##### 3.2.2.6. Free-response section:

The three free-response questions are shown below together with their answers:

- What for has NAO been most useful?:
  − Student 1: To encourage me.
  − Student 2: To motivate me.
- What did you liked most in the experience?:
  − Student 1: Practicing with Scratch.
  − Student 2: When MONICA was trying to be funny.
- What did you liked less in the experience?:
  − Student 1: That MONICA did not helped me more.
  − Student 2: When MONICA gave inappropriate answers.

### 3.3. Classification model

For the data training, we have used the cross validation method with five-folds, where four of these five-folds were used to train the algorithm, and the last one was used as a test set.

The Table 10 summarizes the AUC/ROC (Area Under the Curve/Receiver Operating Characteristic) results obtained in each of the classification method.

**Table 10:** **AUC/ROC values for the methods used**.

	**C4.5**	**K-NN**	**Neural Networks**	**SVM**	**Nave Bayes**
Concentrating	0.779	0.831	0.818	0.769	0.815
Distracted	0.861	0.942	0.883	0.864	0.946
Inactive	0.813	0.861	0.858	0.809	0.856
Mean	0.799	0.852	0.84	0.793	0.847

The ROC graph is a technique for viewing, organizing and selecting classifiers based on their performance. The ROC curve has an interesting property: although the distribution of the data changes, this curve does not change, so it is independent from the distribution of the data, unlike other measurements (for example the Recall curve).

To compare this curve for the different classifiers, we can reduce to a scalar value (between 0 and 1), calculating the existing area under the curve (called Area Under the Curve or AUC), which is equivalent to the probability that the classifier scores higher a positive random instance than a negative random instance.

Based in the mean of the AUC/ROC we have, in order from best to worst results:

1. K-NN: 0.852
2. Naïve Bayes: 0.847
3. Artificial Neural Network: 0.84
4. C4.5: 0.799
5. SVM: 0.793

In this respect K-NN has better results. However in one hand this method has trained with a k value based in the dataset (the k value with a minor error), making without having a larger dataset to prove it, fits the possibility of having committed an overfitting. In the other hand, K-NN is a lazy method and has the disadvantage that the prediction can be slow and depends of the example set.

Note that the use of Support Machine Vector has been unprecedented in this kind of application, and although it has been the method with worst results, these results have come closer to the best results obtained, which enables it to potential future studies within this kind of application.

Finally, for the model generation, the Naïve Bayes method is used with the kernel estimator, as it has presented a good performance with continuous variables, as well as good results in the AUC/ROC, and the parameterization is minimal and independent of the dataset.

### 3.4. MONICA evaluation

The results of the assessment of the implemented prototype MONICA have not been very good, due to factors such as that the prototype was very basic and we have had just two children for the experience.

However, these results have provided feedback for future work, our main conclusions being:

- The playful aspect of the robot (sense of humor) is a very important motivating factor.
- Correct parametrization of the pedagogical intervention model is important in order to avoid causing frustration to the students carrying out the activities.
- Regarding the pedagogical intervention, it is also important to implement a proper Learning Scenario component that gives the robot the capacity to identify the learning scenarios that cause most difficulties in each of the proposed activities.
- The student model is just as crucial. Thus the simple model used do not include the age of the students, factor which our experiment has shown relevant (thus, the youngest girl lost more easily her patience and had a different sense of humor).
- Another element that can cause much frustration to the students is the misidentification of cognitive-affective states, which highlights the importance of the Emotional Model component.

## 4. Discussion

In the field of online educational systems, and in particular affective educational recommender systems, no references have been found in the literature about modeling the emotions of primary school pupils, nor about the use of affective robot tutors together with low-cost and non-intrusive methods for detecting the emotional states of learners. The availability of non-intrusive and low-cost methods and tools to facilitate social interaction between children and robots is crucial to the use of affective robot tutors becoming widespread in infant and primary education. Furthermore, the robot tutors lack the data from the student's interaction with the computer which an affective educational software can access to diagnose affective states (mainly keyboard and mouse inputs and facial images which are easy to interpret when the student looks at the screen).

Moreover, to operate as a genuine tutor and make appropriate educational interventions, a robot must know the learning environment (course contents, competencies of students, …). A possible means to provide a robot with this knowledge is to establish a communication between the robot and an affective educational recommender system with which the students interact. However, in the educational context, we have not found any reference in the literature about robots which operate jointly with an educational recommender system.

In this research we have found it to be of great interest to examine the use of keyboard and mouse interaction data in the particular case of primary school students. To that end, we carried out some experiments in which we found as a first approximation, three cognitive-affective states: concentrating, distracted and inactive.

Thus, in order to facilitate the implementation of affective robot tutors that communicate with educational software designed for primary school children, and that take account the idiosyncrasies of these students, we have provided an architectural pattern, a development methodology and a software development platform for the development of robot tutors (ARTIE).

The implementation of our prototype using ARTIE and its deployment in the classroom suggests that it is possible to make affective educational interventions adapted to the idiosyncrasies of primary education through the use of a robot tutor.

ARTIE has been validated via a deployment using Scratch as educational software, and NAO as affective robot tutor, providing a framework to define appropriate affective pedagogical interventions according to student models and student emotional states, described using a generic markup language. The ARTIE platform also provided the ability to translate the BML language into NAO actions.

The Emotional Model component integrated in the ARTIE platform identifies, through keyboard and mouse interaction data, those cognitive-affective states of primary school pupils that condition the character of educational interventions. Given the non-intrusive nature of our identification method, the students keep their focus on the learning activities as we observed during our experiment.

During the experimentation of the prototype we also observed that the students interacted naturally with NAO and followed its recommendations while feeling relaxed and motivated. However, we also observed that a robot with very basic teaching and student models, a high number of interventions in a short time, and poor communication abilities may cause frustration to the children. From this fact we conclude that a comprehensive student model and a good pedagogical intervention model, acted out by means of a rich gesture and verbal affective language, adequate to every specific learning scenario for each educational software are of critical importance. Regarding the pedagogical intervention, giving the robot the capacity to identify the learning scenarios that cause most difficulties to students is also critical. Another element that can cause much frustration to the students is the misidentification of cognitive-affective states.

The results of our experiment have also provided feedback for future work by revealing the playful aspect of the robot as a very important motivating factor.

Most of the components of the ARTIE platform are currently quite rudimentary and should be further developed in future versions. Our aim is to equip the platform with libraries of both emotion detection methods, translators of BML to robot actions for a variety of commercial robots, and intervention pedagogical models of different educational approaches, as well as with a comprehensive database of recurrent sentences, jokes, etc. with which to build dialogues. Our purpose is also to keep on experimenting with new emotion detection methods tailored to the characteristics of primary school students. As reported on in this paper, for the moment we have experimented with the SVM method, which have no precedents in the literature of the field, obtaining quite positive results.

The ARTIE methodology for designing experiences to elicit the educational intervention expertise has proven adequate in the primary education context. Despite this, the methodology still needs to be defined in more detail.

Our experience has particularly shown the potential of ARTIE as a tool for easily turning any affective educational recommender system in which an avatar plays the role of an affective virtual tutor, into an educational system in which student learning is supported by a robot tutor.

## Author contributions

LI Research project design; Architectural Design, implementation, and experimentation, state of the art; article editing ÁM Research project design, Educational systems consulting, state of the art, validation of the experimental results, article editing. FD Robotics consulting, state of the art and validation of the experimental results.

### Conflict of interest statement

The authors declare that the research was conducted in the absence of any commercial or financial relationships that could be construed as a potential conflict of interest.

## References

[B1] Alimisis D. (2013). Educational robotics: open questions and new challenges. Themes Sci. Technol. Educ. 6, 63–71.

[B2] Bailenson J. N. Yee N. Brave S. Merget D. Koslow D. (2007). Virtual interpersonal touch: expressing and recognizing emotions through haptic devices. Hum. Comput. Interact. 22, 325–353. 10.1080/07370020701493509

[B3] Beard S. Gustavsson C. Strindlund L. Wiknertz E. Huynh Q. Marriott A. (2001). VHML. Available online at: http://www.vhml.org/documents/VHML/2001/WD-VHML-20011021/vhml.pdf

[B4] Bertola F. Patti V. (2013). Organizing artworks in an ontology-based semantic affective space. ESSEM@AI^*^IA 1096, 119–130.

[B5] Blazar D. (2015). Attending to General and Content-Specific Dimensions of Teaching: Exploring Factors Across Two Observation Instruments. Cambridge, MA: National Center for Teacher Effectiveness.

[B6] Bradley M. Lang P. J. (1994). Measuring emotion: the self-assessment semantic differential. J. Behav. Ther. Exp. Psychiatry 25, 49–59. 7962581 10.1016/0005-7916(94)90063-9

[B7] Breazeal C. Aryananda L. (2002). Recognition of affective communicative intent in robot-directed speech. Auton. Robots 12, 83–104. 10.1023/A:1013215010749

[B8] Capponi M. F. Nussbaum M. Marshall G. Lagos M. E. (2010). Pattern discovery for the design of face-to-face computer-supported collaborative learning activities. J. Educ. Technol. Soc. 13, 40–52.

[B9] Catlin D. Robertson S. (2012). Using educational robots to enhance the performance of minority students, in TRTWR 2012 Conference (Riva La Garda), 12–21.

[B10] Choi J. Cho Y. Choi J. Choi J. (2014). A layered middleware architecture for automated robot services. Int. J. Distrib. Sens. Netw. 2014:201063 10.1155/2014/201063

[B11] Chuttur M. (2009). Overview of the technology acceptance model: origins, developments and future directions. Sprouts 9, 1–23.

[B12] Cocea M. Weibelzahl S. (2009). Log file analysis for disengagement detection in e-Learning environments. User Model. User Adapt. Interact. 19, 341–385. 10.1007/s11257-009-9065-5

[B13] Fong T. Nourbakhsh I. Dautenhahn K. (2002). A survey of socially interactive robots. Rob. Autonom. Syst. 42, 143–166. 10.1016/S0921-8890(02)00372-X

[B14] Fridin M. Belokopytov M. (2013). Computers in human behavior acceptance of socially assistive humanoid robot by preschool and elementary school teachers. Comput. Hum. Behav. 33, 23–31. 10.1016/j.chb.2013.12.016

[B15] Gascueña J. M. Fernández-caballero A. González P. (2004). Ontologías del modelo del alumno y del modelo del dominio en sistemas de aprendizaje adaptativos y colaborativos. Available online at: http://www.info-ab.uclm. es/personal/AntonioFdez/download/papers/conference/INTERACCION2005-ontomo delos.pdf

[B16] Gocłowska B. Kufel M. Puchacz M. Świech R. (2007). Rule Languages Used in Tutorial Chatbots Programming. Vol. 7 Lublin: Wydawn, Uniwersytet Marii Curie-Sklodowskiej.

[B17] Grassi M. (2009). Developing HEO Human Emotions Ontology, in Lecture Notes in Computer Science (Including Subseries Lecture Notes in Artificial Intelligence and Lecture Notes in Bioinformatics), 5707 LNCS (Ancona: Università Politecnica delle Marche), 244–251.

[B18] Hammer S. Rist T. Kastrinaki S. Hondrou C. Raouzaiou A. Karpouzis K. (2015). Design of a lifestyle recommender system for the elderly : requirement gatherings in Germany and Greece, in PETRA '15 Proceedings of the 8th ACM International Conference on PErvasive Technologies Related to Assistive Environments (New York, NY: ACM).

[B19] Hastings J. Ceusters W. Mulligan K. Smith B. (2012). Annotating affective neuroscience data with the emotion ontology, in Proceedings of the Workshop Towards an Ontology of Mental Functioning, ICBO 2012 (Graz), 1–5.

[B20] Hastings J. Ceusters W. Smith B. Mulligan K. (2011). The emotion ontology: enabling interdisciplinary research in the affective sciences, in Lecture Notes in Computer Science (including subseries Lecture Notes in Artificial Intelligence and Lecture Notes in Bioinformatics), 6967 LNAI (Geneva: University of Geneva), 119–123.

[B21] Hernández Orallo J. Ramrez Quintana M. Ferri Ramrez C. (2004). Introducción a la Minerde Datos. Pearson Prentice Hall.

[B22] Hernando A. (2015). Viaje a la escuela del siglo XXI.

[B23] Heylen D. Kopp S. Marsella S. Vilhjalmsson H. (2008). The next step towards a functional markup language, in International Workshop on Intelligent Virtual Agents (Berlin; Heidelberg: Springer-Verlag), 270–280.

[B24] Ismail L. I. Shamsudin S. Yussof H. Hanapiah F. A. Zahari N. I. (2012). Estimation of concentration by eye contact measurement in robot based intervention program with autistic children. Proc. Eng. 41, 1548–1552. 10.1016/j.proeng.2012.07.348

[B25] Jimenez F. Yoshikawa T. Furuhashi T. Kanoh M. (2015). An emotional expression model for educational-support robots. J. Artif. Intell. Soft Comput. Res. 5, 51–57. 10.1515/jaiscr-2015-0018

[B26] Kalelioglu F. Gülbahar Y. (2014). The effects of teaching programming via Scratch on problem solving skills: a discussion from learners' perspective. Inform. Educ. 13, 33–50.

[B27] Kamei K. Ikeda T. Kidokoro H. Shiomi M. Utsumi A. Shinozawa K. (2011). Effectiveness of cooperative customer navigation from robots around a retail shop, in Proceedings - 2011 IEEE International Conference on Privacy, Security, Risk and Trust and IEEE International Conference on Social Computing, PASSAT/SocialCom 2011 (Kyoto), 235–241.

[B28] Kaur G. Chaudhary D. (2015). Semantic web : a boon for E-learning. Int. J. Adv. Res. Comput. Commun. Eng. 4, 484–486.

[B29] Keren G. Ben-David A. Fridin M. (2012). Kindergarten assistive robotics (KAR) as a tool for spatial cognition development in pre-school education, in IEEE International Conference on Intelligent Robots and Systems (Vilamoura), 1084–1089.

[B30] Khan I. A. Brinkman W. P. Hierons R. (2013). Towards estimating computer users' mood from interaction behaviour with keyboard and mouse. Front. Comput. Sci. 7, 943–954. 10.1007/s11704-013-2331-z

[B31] Khanna P. Sasikumar M. (2010). Recognising emotions from keyboard stroke pattern. Int. J. Comput. Appl. 11, 1–5. 10.5120/1614-2170

[B32] Koo S.-Y. Park K. Kim H. Kwon D.-S. (2011). A dual-layer user model based cognitive system for user-adaptive service robots, in Symposium A Quarterly Journal In Modern Foreign Literatures (Atlanta, GA), 59–64.

[B33] Kopp S. Krenn B. Marsella S. Marshall A. Pelachaud C. Pirker H. (2006). Towards a common framework for multimodal generation: the behavior markup language. Intell. Virtual Agents 4133, 205–217. 10.1007/11821830_17

[B34] Liu I. F. Chen M. C. Sun Y. S. Wible D. Kuo C. H. (2010). Extending the TAM model to explore the factors that affect Intention to Use an Online Learning Community. Comput. Educ. 54, 600–610. 10.1016/j.compedu.2009.09.009

[B35] Liu W. Dong W. (2015). Research on improved dialogue model. Int. Conf. Educ. Technol. Manage. Human. Sci. 827–831. 10.2991/etmhs-15.2015.182

[B36] Lohse M. Welbergen H. V. (2012). Designing appropriate feedback for virtual agents and robots, in … Robot Feedback Human-Robot Interaction, Vol. 12.

[B37] López García J. C. (2012). Identificación y regulación de emociones con Scratch, in Tendencias Emergentes en Educación Con TIC (Asociación Espiral, Educación y Tecnología), 67–81.

[B38] Manjarrés-Riesco Á. Santos O. C. Boticario J. G. (2013). Eliciting affective recommendations to support distance learning students, in User Modeling, Adaptation, and Personalization, eds Carberry S. Weibelzahl S. Micarelli A. Semeraro G. (Berlin; Heidelberg: Springer), 347–349.

[B39] Meftah I. T. (2013). Modélisation, Détection et Annotation des États Émotionnels á L'aide d'un Espace Vectoriel Multidimensionnel. Docteur en Sciences Tayari Meftah, Université Nice Sophia Antilpolis. Available online at: https://hal-unice.archives-ouvertes.fr/file/index/docid/908233/filename/130412_manuscrit_final_I-Tayari.pdf

[B40] Mubin O. Stevens C. J. Shahid S. Mahmud A. A. Dong J.-J. (2013). A review of the applicability of robots in education. Technol. Educ. Learn. 1, 209–215. 10.2316/Journal.209.2013.1.209-0015

[B41] Nozawa Y. Dohi H. Iba H. Ishizuka M. (2004). Humanoid robot presentation controlled by multimodal presentation markup language MPML, in RO-MAN 2004. 13th IEEE International Workshop on Robot and Human Interactive Communication (IEEE Catalog No.04TH8759) (Tokyo), 153–158.

[B42] Ocumpaugh J. (2012). Baker-Rodrigo Observation Method Protocol (BROMP) 1.0.

[B43] Ocumpaugh J. College T. (2014). Baker Rodrigo Ocumpaugh Monitoring Protocol (BROMP) 2.0 Technical and Training Manual.

[B44] Paiva A. Leite I. Ribeiro T. (2012). Emotion Modelling for Social Robots. Oxford: Oxford University Press.

[B45] Pardo X. C. Martin M. J. Sanjurjo J. Regueiro C. V. (2009). Teaching digital systems in the context of the new european higher education area : a practical experience. IEEE Trans. Educ. 52, 513–523. 10.1109/TE.2008.930512

[B46] Pellegrini A. D. Blatchford P. (2000). The Child at School: Interactions with Peers and Teachers. Springer.

[B47] Picard R. Vyzas E. Healey J. (2001). Toward machine emotional intelligence: analysis of affective physiological state. IEEE Trans. Pattern Anal. Mach. Intell. 23, 1175–1191. 10.1109/34.954607

[B48] Porayska-Pomsta K. Mavrikis M. D'Mello S. Conati C. Baker R. S. J. D. (2013). Knowledge elicitation methods for affect modelling in education. Int. J. Artif. Intell. Educ. 22, 107–140. 10.3233/JAI-130032

[B49] Prendinger H. Ullrich S. Nakasone A. Ishizuka M. (2011). MPML3D: Scripting agents for the 3D internet. IEEE Trans. Vis. Comput. Graph. 17, 655–668. 10.1109/TVCG.2010.66 20479495

[B50] Rajput S. Vijayavargiya P. (2015). Objective of keystroke dynamics for identifying emotional state. Int. J. Comput. Sci. Info. Technol. 6, 632–636.

[B51] Resch B. Sudmanns M. Sagl G. Summa A. Zeile P. Exner J.-P. (2015). Crowdsourcing physiological conditions and subjective emotions by coupling technical and human mobile sensors, in GI_Forum 2015 - Geospatial Minds for Society, Vol. 1 (Verlag der Österreichischen Akademie der Wissenschaften), 514–524.

[B52] Ribeiro T. Pereira A. Deshmukh A. Aylett R. Paiva A. (2013). I'm the Mayor: a robot tutor in Enercities-2 (demonstration) in Proceedings of the 2014 International Conference on Autonomous Agents and Multi-Agent Systems (Paris), 1675–1676.

[B53] Riggs N. R. Greenberg M. T. Kusché C. A. Pentz M. A. (2006). The mediational role of neurocognition in the behavioral outcomes of a social-emotional prevention program in elementary school students: effects of the PATHS curriculum. Prev. Sci. 7, 91–102. 10.1007/s11121-005-0022-1 16572300

[B54] Robins B. Dautenhahn K. Dubowski J. (2004). Investigating autistic children's attitudes towards strangers with the theatrical robot - a new experimental paradigm in human-robot interaction studies, in RO-MAN 2004. 13th IEEE International Workshop on Robot and Human Interactive Communication (IEEE Catalog No.04TH8759) (Hertfordshire), 557–562.

[B55] Saerbeck M. Schut T. Bartneck C. Janse M. D. (2010). Expressive robots in education varying the degree of social supportive behavior of a robotic tutor, in Proceedings of the SIGCHI Conference on Human Factors in Computing Systems (ACM), 1613–1622.

[B56] Salam H. Chetouani M. (2015). A multi-level context-based modeling of engagement in human-robot interaction, in 11th IEEE International Conference and Workshops on Automatic Face and Gesture Recognition (FG), 2015 (IEEE), 1–6.

[B57] Salmeron-majadas S. (2014). Affective standards-based modeling in educational contexts from mining multimodal data sources State of the art in UMAP Workshops.

[B58] Santos O. C. Boticario J. G. (2011). Tormes methodology to elicit educational oriented recommendations, in Artificial Intelligence in Education, eds Biswas G. Bull S. Kay J. Mitrovic A. (Berlin; Heidelberg: Springer), 541–543.

[B59] Santos O. C. Boticario J. G. Manjarrés-Riesco Á. (2014). An approach for an affective educational recommendation model, in Recommender Systems for Technology Enhanced Learning, eds Manouselis N. Drachsler H. Verbert K. Santos O. C. (New York, NY: Springer), 123–143.

[B60] Selim H. M. (2003). An empirical investigation of student acceptance of course websites. Comput. Educ. 40, 343–360. 10.1016/S0360-1315(02)00142-2

[B61] Shelton C. D. (2012). Robot-Object Interaction Language. Auburn University.

[B62] Softbank Robotics (2016). Available online at: https://www.ald.softbankrobotics.com/en/cool-robots/nao/find-out-more-about-nao

[B63] Tanaka F. Matsuzoe S. (2012). Children teach a care-receiving robot to promote their learning: field experiments in a classroom for vocabulary learning. J. Hum. Robot Interact. 1, 78–95.

[B64] Tang D. Yusuf B. Botzheim J. Kubota N. Chan C. S. (2015). A novel multimodal communication framework using robot partner for aging population. Expert Syst. Appl. 42, 4540–4555. 10.1016/j.eswa.2015.01.016

[B65] Tsutsui T. Saeyor S. Ishizuka M. (2000). MPML : A Multimodal Presentation Markup Language with Character Agent Control Functions Features of Multimodal Presentation Markup Language MPML, in WebNet, 537–543.

[B66] Valle J. E. M. Salgado V. C. (2013). Pensamiento lógico matemático con scratch en nivel básico. Vínculos 9, 87–95.

[B67] Veena Vijayan V. (2014). Investigation about the impact of robots in educational and medical field. Int. J. Comput. Sci. Mobile Comput. 3, 436–441.

[B68] Vouloutsi V. Munoz M. B. Grechuta K. Lallee S. Duff A. Llobet J.-y. P. (2014). A new biomimetic approach towards educational robotics : the distributed adaptive control of a synthetic tutor assistant.

[B69] Wilson A. Moffat D. C. (2010). Evaluating Scratch to introduce younger schoolchildren to programming, in Proceedings of the 22nd Annual Workshop of the Psychology of Programming Interest Group (Glasgow), 64–75.

[B70] Zimmermann P. Guttormsen S. Danuser B. Gomez P. (2003). Affective computing– a rationale for measuring mood with mouse and keyboard. Int. J. Occup. Saf. Ergon. 9, 539–551. 10.1080/10803548.2003.11076589 14675525

